# A phenomenological exploration of self-identified origins and experiences of body dysmorphic disorder

**DOI:** 10.3389/fpsyg.2022.963810

**Published:** 2022-09-23

**Authors:** Shioma-Lei Craythorne, Rachel L. Shaw, Michael Larkin

**Affiliations:** Institute of Health and Neurodevelopment, Aston University, Birmingham, United Kingdom

**Keywords:** body dysmorphia, interpretative phenomenological analysis, bullying, qualitative research, BDD origins, BDD development

## Abstract

Body dysmorphic disorder (BDD) is a debilitating mental health condition that presently affects ~2% of the general population. Individuals with BDD experience distressing preoccupations regarding one or more perceived defects in their physical appearance. These preoccupations and perceived distortions can have a profound impact on key areas of social functioning and psychological health. Individuals’ BDD origins have not been explored in significant depth and have been, often unhelpfully, conflated with social media usage and exposure to idealistic imagery of the body. Such generalisations fail to acknowledge the complexity of BDD development and onset, highlighting the importance of moving towards an understanding of people’s implicit theories regarding their own experience. It is therefore essential to gain insight into how individuals make sense of the experiences which they believe led to the development and onset of BDD. The aim of this exploratory study was to elicit and phenomenologically analyse the accounts of individuals with lived experience of BDD in order to examine their beliefs about its origins and understand how they navigate the world with a distorted sense of self. Participants provided written and verbal accounts regarding both their BDD onset and experiences of living with the disorder. Both components of the study were analysed using Interpretative Phenomenological Analysis. Four main themes were generated from the data: *Exposure to bullying and external critique of appearance*; *Experiencing rejection, shame, and a sense of not being enough*; *Developing an awareness of the solidification of concerns*, and *Learning about and reflecting upon triggers*. Participants attributed their BDD onset to adverse experiences such as childhood bullying, receiving appearance-focused criticism, rejection and being subjected to emotional and physical abuse. The findings from this study highlight the complexity of BDD development and onset in individuals, and the need for appropriate care and treatment for those affected by BDD.

## Introduction

Body Dysmorphic Disorder (BDD) is a debilitating mental disorder, characterised by “a distressing or impairing preoccupation with an imagined or slight defect in appearance” (Ruffolo et al., 2006: 11). The prevalence of the disorder is thought to range from 0.7% to 2.4% within the general population (Faravelli et al., 1997; Phillips, 2005; Kelly et al., 2010) and can affect people of varying ages. Typically, the average age at onset is during adolescence, at around 16 years of age (Bjornsson et al., 2013), but the root causes of the disorder remain unknown. At present, it can take up to 15 years to receive a clinical diagnosis for BDD (Veale et al., 2016: 183). Many people living with it are misdiagnosed with depression, social anxiety or social phobia (Phillips, 2005: 40), and therefore do not receive appropriate treatment. Often, people living with BDD are too ashamed to seek help, largely due to the fear of being perceived as vain (Phillips, 2005; Buhlmann et al., 2009). Recent findings regarding suicidality in BDD also discovered that people living with BDD were four times more likely to have experienced suicidal ideation and 2.6 times more likely to have attempted suicide than individuals diagnosed with eating disorders, Obsessive Compulsive Disorder (OCD) or other anxiety disorders (Angelakis et al., 2016: 61). It is therefore important to increase awareness of this under-researched disorder, and amplify the voices of people who live with it. Accounts of the lived experience of people living with BDD and its origins are lacking in academic research, and it is essential to understand the condition better through people’s experiences, helping to provide earlier diagnosis and design acceptable and appropriate services. In order to do this, we must understand how it can develop and in what circumstances. With regard to the identification of potential contributing factors of BDD development, this is still an area that remains unclear and under-researched across the literature. It should be noted that, as yet, there is no definitive set of events that are ‘proven’ to contribute to the development of BDD. Researchers have suggested that there may be numerous predisposing factors that have influence over the development, or triggering, of the condition. Some of these proposed factors include genetic or hereditary implications and physical differences in brain structure, such as differences in regional brain volumes (Buchanan et al., 2014), and reduced cortical thickness (Grace et al., 2017) in comparison to healthy control groups. Neziroglu et al. (2004) proposed a biopsychosocial model for the examination of the presence of environmental influences of BDD development. Their biopsychosocial model takes into account external stimuli including cultural beliefs and values, life events and social influences (Neziroglu et al., 2004) as well as the neurobiological and evolutionary factors that may be associated with the development of BDD. According to this model, some of the key social and environmental influences or risk factors that may lead to or trigger BDD include pressures from society to be ‘perfect’ due to cultural beauty standards, and aesthetic sensitivity.

Despite emerging interest in BDD research, the majority of the research is quantitative. Therefore, there is still very little understanding of the personal, idiographic experiences of those living with the disorder. Of the qualitative BDD research available, studies have found that there are notable inconsistencies between the ideal and actual selves of those living with the condition (Silver et al., 2010; Silver and Reavey, 2010; Brohede et al., 2016). Silver and Farrants (2015) conducted a qualitative study exploring BDD participants’ experiences of mirror gazing using a combination of photo elicitation and Interpretative Phenomenological Analysis (IPA; Smith, 1996). Their study considered the motivations of individuals with BDD who engaged in mirror gazing and provided rich insight into the phenomenon; however, the study focused on one specific manifestation of BDD, rather than exploring multiple facets of the disorder. Weingarden et al. (2017) published a mixed-methods study investigating triggering events that patients believed contributed to their development of BDD. This study involved analysing the self-identified triggering factors of 165 adult BDD patients, and it was suggested that the most common events contributing to the emergence of BDD symptoms described in their narratives were experiencing bullying, abuse and family stress. Although this study collected data from a large sample size and gave a good idea of the scope of events people with BDD may attribute to the disorder, it did not explore the significance in meaning of those events to the individuals. For example, the descriptor ‘family stress’ is vague, and it is unclear what the respondents’ experience of family stress involved, and how they believe it may have triggered their BDD.

To gain more clarity with regard to the lived experience of BDD and what individuals believe the origins of the disorder to be, we have drawn on concepts of phenomenology and psychological literature to make sense of our findings. Phenomenology is “a philosophical approach to the study of experience” (Smith et al., 2022: 7). We employed Interpretative Phenomenological Analysis (IPA; Smith, 1996) to “understand and ‘give voice’ to the concerns of participants” (Larkin et al., 2006: 102). To the best of our knowledge, this is the first paper to use IPA as a methodological approach to explore the accounts of people living with BDD regarding both its possible origins and experiences of living with it. Through our analysis, we generated four main themes: *Exposure to bullying and external critique of appearance*; *Experiencing rejection, shame, and a sense of not being enough*; *Developing an awareness of the solidification of concerns*, and *Learning about and reflecting upon triggers*.

## Materials and methods

### Approach and design

In this study, we used Interpretative Phenomenological Analysis (IPA; Smith, 1996). IPA is a methodological approach used to gain deep insight into the lived experiences of people, with a focus on how they experience and make sense of phenomena. This study is a qualitative and exploratory design with a phenomenological focus. Phenomenology is concerned with “describing the world as it appears to people” (Langdridge, 2007: 11), and is therefore a useful approach to consider the way in which we understand the world through our own perception. In this study, we were interested in finding out how people made sense of the origins of their BDD. In particular, we were interested in identifying instances where participants reflected on their experiences and reflections with regard to their earliest memories of their BDD development.

### Sampling strategy and recruitment

We sought to recruit participants who were aged 18 or over, and who identified as living with BDD. The recruitment process began once ethical approval was gained from the Aston University Health and Life Sciences Research Ethics Committee (Reference number: 1355). The first author circulated a study advertisement on Twitter. The advertisement was also shared by the Body Dysmorphic Disorder Foundation (BDDF) on Twitter and on their website.^1^ Interested participants were invited to contact the first author *via* email to request a participant information sheet and link to a screening questionnaire hosted by Qualtrics containing a box for them to confirm they were aged 18 years or over, the Body Dysmorphic Disorder Questionnaire (BDDQ) and a consent form. The BDDQ was used as a tool to screen in people with BDD. It asks participants structured questions about how they feel about their appearance at the time of completion.

### Participants

Sixteen potential participants responded to the advertisements. Of those 16 participants, eight subsequently took part in both components of the study.

Inclusion was not dependent on a clinical diagnosis but participants’ own identification as someone living with BDD, given the difficulty in gaining a diagnosis. Some participants were in the process of being referred for diagnosis at the time of their interview.

Two participants identified as male and six as female. Six participants lived in the United Kingdom, one in the United States and one in India.

### Data collection procedure

Data collection consisted of two parts: a biographical writing task and an interview.

#### Biographical writing task

First, we asked participants to complete a biographical writing task in which they were asked to write about the first time they experienced negative feelings about their appearance. They were emailed the same question and prompts to help them answer the question. Answering the prompting questions was optional, and the structure of the written piece was entirely at the discretion of the participant.

Participants who were eligible to take part in the study were sent an email from the first author within 3 days of completing the screening questionnaire, inviting them to answer the following question in their biographical writing task:

*Can you tell me about a time when you first became worried about your appearance?*

*Some other prompts to consider:*

- *What was going on in your life at the time?*
- *Did you speak to anyone about it at the time? If you did, who did you speak to?*
- *How were you feeling?*
- *What were you thinking?*
- *What did you do about it?*

Eliciting written descriptions from participants prior to conducting interviews enabled us to gain insight into their individual worlds to then facilitate the interview that followed. Using experiential material, such as the combination of written descriptions and interviewing permitted us to move away from the dominant use of interviews alone, frequently used in qualitative research.

#### Interview

Once participants had completed their writing tasks, we invited them to take part in a follow-up semi-structured interview to explore what they had written in more depth. A semi-structured interview follows an interview guide, “which structures the course of the interview more or less tightly” (Kvale and Brinkmann, 2009: 130). Semi-structured interviews are commonly used in IPA studies as a way of collecting data (Smith, 2016), and are developed to be flexible and led by participants.

The first author conducted all of the interviews. They lasted, on average, 68 min. Participants were given a choice of interview media (see Table 1). Eight participants completed both the biographical writing task and interview components of the study. Two participants requested their interviews were conducted *via* email, and the remaining six were conducted *via* Skype audio, telephone or Skype video.

**Table 1:** Participant demographics.

Pseudonym	Age at interview	Sex	Location	Interview medium
Angelina	28	F	USA	Email
Bethany	53	F	UK	Telephone
Claire	32	F	UK	Telephone
Elliot	44	M	UK	Telephone
Jenny	34	F	UK	Skype (Video)
Kate	20	F	UK	Telephone
Rohit	28	M	India	Skype (Audio)
Victoria	18	F	UK	Email

We based our semi-structured interviews on the content of participants’ biographical writing tasks. This meant participants had some control over the content of the interview. The interviewer reminded participants that they did not have to answer any questions they were not comfortable answering. In line with the flexibility of semi-structured interviews, the interviewer asked questions directly related to the accounts participants provided in their biographical writing tasks and adapted the order of questions. Once questions about the writing task had been asked, the interviewer also asked questions about their current experiences of living with BDD for additional context (Table 2).

**Table 2:** Indicative interview topic guide.

Example questions	Prompts
In your written description, you told me about [x]. Please could you tell me more about this?	How were you feeling when you were going through this? What was that like? What support did you have at that time? How were you feeling when you wrote about this? How do you feel about this now?
(In a face-to-face interview/ Skype video interview) If I had not met you, and we had only spoken on the phone, how would you describe your appearance?	How do you feel when you think about your appearance? What do you like about your appearance? What was it like to describe your appearance?
If I asked your friends, how would they describe your appearance?	What descriptors would you agree/disagree with and why? What support do your friends offer? How do you feel when they do/say this? What do you think your friends see when they look at you?
Could you describe how your feelings about your appearance have changed over time?	Have your thoughts changed? How? Do you do anything in your daily routine that changes the way you feel about your appearance? Would you change anything? What do you like the most about your appearance?

### Data analysis procedure

The first author transcribed each audio interview verbatim and assigned pseudonyms to all names of people and places mentioned. When coding the data, we were particularly interested in participants’ use of figurative and descriptive language with regard to understanding what they identified as contributing factors towards the development of their BDD and how they made sense of their experiences, as recommended by Smith et al. (2022). The first author coded the data, and the codes were discussed in supervision with the second and third authors. The first author proposed the initial thematic structure, which we discussed and further developed through supervision.

## Analysis

Through our analysis, we generated four themes: Being exposed to bullying and critique of appearance; Experiencing rejection, shame and a sense of not being good enough; Becoming aware of the solidifying of concerns; and Learning about and reflecting on triggers. The first theme of interest was ‘Being exposed to bullying and critique of appearance’, which caused great distress to a number of participants in the study.

### Exposure to bullying and external critique of appearance

Experiencing appearance-focused bullying and criticisms of appearance from a young age was reported by participants as a potential origin of their BDD. The criticisms were often made by peers, friends and family members, and in most cases, the aspect of the appearance that received the criticism went on to become an area of concern and distress for the participant. Criticisms were often extensive and were not limited to one aspect of the participants’ appearance.

Such criticisms were experienced by Angelina, who shared her experience of being bullied during her childhood and discussed how the same criticisms of her appearance continue to affect her in adulthood.

It was around the time of 3^rd^ or 4^th^ grade [aged approximately 8–9 years] that I started to experience bullying, which usually consisted of insults and ridicule directed towards my appearance. I was literally verbally “attacked” from head to toe; my hair, my nose, my moles, my body hair, my weight and size, my “lack of curves”… Even though I tried to hide it and act unbothered by them, these comments cut me to the core and I stored them deep within the corners of my brain, ruminating on them even to this day. (Angelina, Biographical writing task)

The criticisms she received from her peers targeted numerous aspects of her face and body, and were extensive and unremitting in nature. The amount of criticisms she received may have contributed to her feeling overwhelmed by the negative perceptions other people had ascribed to her body. By using evocative words such as “insults,” “ridicule” and “attacked,” Angelina helps us to realise how adverse these incidents of bullying were for her. She described hiding her true feelings towards what the bullies said to her at the time by pretending that their comments did not bother her; however, her distress was palpable as she revealed through a powerful metaphor that they “cut [her] to the core.” The word ‘cut’ demonstrates just how damaging those criticisms were to Angelina, and how, in her view, the words of her bullies were weaponised. Her words “I stored them deep within the corners of my brain, ruminating on them even to this day” convey the tormenting reverberations of the painful bullying experience she endured, and continues to endure. Bullying was also considered a contributing factor towards BDD development by Kate.

Well I’ve-I’ve-I’ve been bullied since I was in year three up until year eleven [aged approximately 7-16 years]… I would have people telling me I look weird… I had people anonymously messaging me and saying that I look like a dog… saying that I’m ugly… I felt hurt that people would actually say it… but at the same time I was like ‘yeah you’re right’… ‘yeah I know I’m that – I just wish I wasn’t’ (Kate, Interview)

The comments Kate received from her peers occurred over an 8-year period of bullying. Being told that she looked ‘weird’, ‘like a dog’ and ‘ugly’ likely instilled the belief within her that something was truly wrong with her appearance, further illustrated by her coming to accept this and agreeing with the comments made. In a similar way to Angelina and Kate, Elliot experienced others commenting on his appearance at a very young age and described a scenario that took place at his school.

When I was at school, aged approximately seven, I was sitting at a table with another boy… and two girls… one of the girls said something like ‘you have lots of beauty spots’ in a pleasant way referring to facial moles I had/have… [the boy], who also had facial moles, said something like ‘they’re not beauty spots, they’re moles’ in a very harsh and disparaging way… I don’t recall openly reacting in any specific way. But I think it was quite soon after that I tried to dig a mole on my right forearm out with a sharp stone in the playground… I also tried to dig a mole out of the rear of my thigh, but that was not successful. (Elliot, Biographical writing task)

Here, Elliot described being in a situation where an aspect of his appearance was brought to the attention of himself and others in a negative and extremely exposing way. He recalled internalising his reaction to a situation that, upon reflection, affected him significantly. Elliot acted on the criticisms of his appearance by engaging in self-surgery in an attempt to remove the aspect of his appearance that made him seem different to others and thus vulnerable to criticism. Using a stone from the playground to perform this action shows how desperate he felt in the situation to find a solution to the negativity he experienced.

In this theme, participants described experiences of being shamed or bullied. They described those experiences as being salient to them years after the incidents had occurred. Participants also perceived themselves as inferior and unequal to the people making the bullying comments and reported that this may have contributed to the development of their BDD.

### Experiencing rejection, shame, and a sense of not being enough

In addition to bullying, experiencing social difficulties in relationships was also thought to be a potential contributing factor towards BDD development. Some participants recalled being exposed to rejection in romantic and familial relationships. The rejection and sense of not being deemed good enough led to an increased concern about appearance and consequently contributed to the development of thoughts associated with BDD. This is illustrated by Claire, who described a scenario in which she experienced rejection from a friend who she considered quite important to her at the time.

I do remember in detail how he teased me that he found my sister attractive and I tried (very poorly on reflection) to flirt with him by stating that ‘I am always told that we both look alike so that must mean that I am attractive to [sic]?’… I distinctly remember the pause, the awkward laugh and the comment ‘No, not really’… it is vivid in my mind during and after the phone call I was staring in the mirror… (Claire, Biographical writing task)

I remember feeling quite um crushed and almost erm taken aback really a bit in shock… it felt like the bottom of my stomach had fallen out (Claire, Interview)

This humiliating experience prompted increased self-scrutiny within Claire, evidenced by her recollection of specific bodily actions such as looking at her reflection in a mirror after the conversation with her friend had ended. She described feeling ‘crushed’, which powerfully illustrates the force the rejection and anguish had on her body. This subsequently led to Claire manifesting these emotions in the form of physical bodily sensations: “it felt like the bottom of my stomach had fallen out.” Another participant, Jenny, also identified a link between experiencing rejection throughout her teenage years and subsequent BDD development.

During the period between the age of 12-16 I remember being rejected by a few boys and I do think that was a contributing factor. Overall, I was heavily comparing myself to others and idolising girls who were slim and pretty. (Jenny, Biographical writing task)

Jenny made a link between being rejected and comparing herself unfavourably to others. In this excerpt, she identified that multiple rejections, in combination with her preconceived notion of what is and is not considered “pretty,” reinforced the perception that she was defective and inadequate compared to her peers. During this time in her life, she described making direct comparisons between her own appearance and the appearance of others she considered to be more desirable. However, not all criticisms and comparisons she reported were related to her appearance. Jenny also faced physical abuse as well as hurtful comments and observations from family members about the way she behaved.

My dad would get short tempered with me and tell me off, although I don't remember the details, I just have a sense of not feeling good enough in his eyes so whatever was said created that belief in me. He once hit me (slapped across my face leaving a hand print) because I did something wrong and I remember the moment clearly, although I have no idea what I did wrong. I also got told repeatedly when I was naughty or annoying that 'I was so much like my auntie [name redacted]' who was viewed very negatively by the family. (Jenny, Biographical writing task)

She described being physically abused by her father for reasons that remain unclear to her, and being directly compared to another family member who was not well liked. In a similar way to Jenny, Rohit experienced many criticisms and comparisons from a family member during his childhood that focused on his behaviours rather than his physical appearance.

She [Rohit’s mother] had an abusive nature, was harshly critical and discouraging towards me. She would keep pointing out mistakes and negatives in me and everything I did. She used foul language and cursed regularly. I do not remember being appreciated or encouraged for anything by her. I remember getting shamed often, in comparison to other kids of my age, my cousins and friends. I received verbal abuse often for the mistakes I made, even simple things like buying the wrong vegetables from the shop. The words were ugly very often. (Rohit, Biographical writing task)

Being shamed and harshly criticised by his mother may have left Rohit vulnerable to developing a poor relationship with his own sense of self during his formative years, which would shape his self-identity as he grew older. Rohit described his mother’s abusive character and reflected on the comparisons drawn with other children his age. We might speculate, by his detailed description, that Rohit’s experience of his mother may have affected his self-concept. Experiencing a lack of appreciation and encouragement from a young age is likely to cultivate negative evaluations about one’s sense of worth. These negative evaluations of the self could then manifest as an overall sense of not being good enough. As the negative manifestations of the sense of self-built up, participants discussed becoming more aware of concerns about aspects of their physical appearance.

### Developing an awareness of the solidification of concerns

Some participants reported having a poor relationship with their appearance from a young age prior to their BDD onset, but this relationship deteriorated further over time and they noticed concerns solidifying and becoming more ‘real’ or concrete to them. This shows one potential way the disorder may develop in some people. It is possible that Victoria may have experienced concerns about her appearance from a young age due to her receiving critical comments about her appearance at school, but describing aspects of her appearance as ‘deformities’ is symptomatic of BDD.

… when I started sixth form I became even more concerned with my appearance and I started to feel as if my flaws were deformities…

I think I found an article about it [BDD] once and it sounded like me or something like that happened I’m not sure. But it sounded like what was happening and I didn’t really know wether [sic] I actually had it or wether [sic] I was being a hypochondriac but I must have it or I’m really hideous and I’m using it as an excuse for my horrible face. It made me feel kind of good that other people had it and I started to read about it more and it would always make me feel sad and I also felt like I didn’t have it because everybody else with it was actually quite pretty and I’m not pretty enough for it to just be my brain lying about a couple of things because it’s not that it’s my actual face being ugly and deformed. (Victoria, Interview)

Victoria appears to express doubt that she has BDD and convinced herself that her perceived flaws were objectively real. She described a tension in her belief of having BDD and not having BDD. The doubt she dwelled upon regarding whether she has BDD appears to be due to the inseparability of her perceived self and objective self. In other words, her perceived distortions are irrefutable, in that she does not consider them to be distortions. She alludes to this idea by using the phrase “my horrible face.” By describing her face as “horrible,” she attaches her distorted perception to her objective appearance, reinforcing their inseparability. She additionally uses words such as ‘hideous’, ‘ugly’, and ‘deformed’ to describe her appearance, conveying how realistic the distortions are to her. Like Victoria, Claire described the foregrounding of her appearance concerns becoming more concrete and problematic during a specific time period.

The longer I looked [in the mirror] the less it made sense what I was looking at… the features seemed to be much more individual rather than looking at a face as a whole…

I don’t see a face as such – I just see faults (Claire, Interview)

Seeing her face as a series of characteristics, or ‘faults’, illustrates the atomistic behaviour that Claire and many others living with BDD experience. Breaking the face down into its constituent parts and not being able to see the whole picture any longer is characteristic of BDD.

Claire momentarily brings our attention to the temporal domain when viewing her appearance in the mirror. She makes a link between the prolonged duration of time spent looking at her appearance and a sense of ambiguity regarding the focus of her gaze she experiences whilst doing so. She described a scenario in which she began to focus on parts of her face in isolation rather than appraising her whole face. This could be interpreted as a gradual process in which she (over a period of time) experienced the separation and possible foregrounding of her perceived problematic features, and a lack of cohesion between them. This supports the idea of appearance concerns solidifying and becoming more apparent to the perceiver over a length of time.

By contrast, Elliot made sense of his BDD onset differently. Unlike other participants, Elliot described the onset of his BDD symptoms as occurring quite suddenly, rather than a gradual increase in tangibility and becoming more real over time. He did not attribute the onset to a particular event or series of events, but he did acknowledge having a problematic relationship with his appearance previously. He spoke about a specific moment in his life when he recalled suddenly not being able to look at a particular aspect of his appearance.

I think BDD really kicked in when I went to university. I think the first real problem I had was looking at the left side of my face. (Elliot, Biographical writing task)

I probably already had-had quite erm an unhealthy relationship with my appearance in a way-but just-it just sort of-I just remember thinking-there was just one day I couldn’t er I just couldn’t cope with looking in the mirror and seeing the left hand side of my face [and] just… just- just-it just sort of – you know – just-just really happened… erm and then it was really-yeah I dunno it just seemed to happen – it wasn’t sort of-I think it may have just been er the overall pressures of being in that situation – not really being… having the resources to deal with it – it wasn’t like – you know – any-it wasn’t- I don’t recall it being anything specific (Elliot, Interview)

Using the phrase ‘kicked in’, suggests Elliot experienced a sudden and notable change in the way he viewed himself. Specifically, the change affected his perception of the left side of his face. Like Claire, Elliot displays the atomistic behaviour characteristic of somebody living with BDD as he identifies the specific area of his face he struggles to view. It is apparent in Elliot’s communication that recalling this significant phenomenon was difficult to express with language. The amount of effort he put into explaining the phenomenon is very apparent with instances of self-correction and hesitancy. Reflecting on his realisation of BDD becoming more apparent to him, Elliot explained: “… one day I couldn’t… cope with looking in the mirror and seeing the left hand side of my face… it just… happened.” His reference to coping in this excerpt is particularly interesting. Elliot may have been able to pinpoint his onset by reflecting on his capacity to cope with appearance-related events leading up to and after that day. It is evident that this day was significant to him, as he is able to recall a time when a part of his life changed, but he cannot express what he believes may have caused it.

Accounts from participants about how they make sense of their appearance concerns solidifying suggest that the rate of BDD onset could vary. It could develop over a period of time or occur quite suddenly. There appears to be a ‘tipping point’ where participants are able to come to a realisation that their relationship with their appearance is not typical, and it may be this point that participants remember more clearly. Some participants were able to identify specific circumstances that made them think about their appearance concerns more often, and this was an important step in their reflective process.

### Learning about and reflecting upon triggers

An important element of understanding the origins of people’s BDD is understanding what people believe their BDD triggers to be. Whilst the triggers may not directly reveal specific origins of the disorder in participants, they help us to gain an understanding of what could worsen BDD in people who live with it, and potentially, how it could be managed. In taking part in the study, participants were able to reflect on the triggers that affect them presently as well as in the past, providing some insight into what they struggle with. Angelina reflected on thoughts she experiences presently with regard to her BDD triggers.

I do have certain triggers that definitely bring them [the thoughts] on, such as if I run into a person I went to school with, if I see a picture of myself that I don’t like or when I look at old pictures; but for the most part the thoughts ARE the triggers and they truly never stop. (Angelina)

Angelina conveys a sense of ambiguity between whether the thoughts she experiences as a result of BDD are triggers or symptoms of BDD. She identified specific events that act as catalysts, leading her to think about the adverse experiences of appearance-focused bullying she went through which subsequently contributed to the development of her negative perception of her appearance. She also acknowledged that her thoughts are continuous throughout her everyday life regardless of the possibility of those triggering events occurring. Like Angelina, Bethany was able to pinpoint specific situations in which her BDD worsens but also highlighted its omnipresence.

My BDD flares up considerably whenever I am stressed or anxious but even when I am not, it is there with me constantly every day… you asked how often I thought about my body image and I can honestly say that it would be impossible to say because it feels like the thoughts are there almost all the time. It would be easier to let you know how often I don't think about it. It is quite often my waking thought and I dream about it regularly. (Bethany, Biographical writing task)

From Bethany’s description, experiencing heightened negative emotions such as stress and anxiousness led her to become more concerned about her perceived flaws in her appearance. However, it is clear that her experience of BDD immerses her lifeworld. Whilst she portrays BDD as being a separate entity, she suggests it is very much fused to her body and self: “*it is there with me constantly every day*.” Due to its perceived permanency, it is difficult for Bethany to identify where BDD begins and ends. It is a constant phenomenon woven throughout her life, and she highlights that it is easier for her to isolate thoughts that are *not* associated with her negative body image.

Victoria was also able to reflect on triggers that worsen her BDD symptoms and identified particular incidents that amplified her negative perception of self. Examples of this included times when her confidence was knocked by a boy (this might include receiving criticisms of appearance or experiencing rejection) or when she, like Angelina, viewed a photograph of herself that she considers unattractive.

When a photo is taken of me I have to spend ages looking at it and it makes me feel disgusting and I feel disgusting now thinking about different photos I’ve looked at with me in. I look deformed and it makes me worry because I must look that vile in real life and I try to look at what individual features are the problem but it’s all of them… it’s something that can’t be fixed easily or can’t be fixed at all. I always notice new features I hate in photos. (Victoria)

Here, Victoria described how viewing a photograph of herself that she considers unattractive can cause elevated and prolonged feelings of worry and concern. Additionally, experiencing a sense of compulsion is conveyed when she explained specific ritualistic behaviours she exhibits: “I have to spend ages looking at it [the photograph].” This demonstrates that she feels compelled to inspect the photographs due to her BDD. She experiences a feeling of disgust if she does inspect a photograph of herself, and this disgust is also evoked by memories of her appearance in photographs too, suggesting she may be haunted by residual distortions. Whilst Victoria was describing her feelings of disgust when viewing photographs in her interview, she said she felt disgusting in that moment “*now* thinking about different photos I’ve looked at with me in” (emphasis added). This could convey how ingrained the images of her perceived self are; she is able to imagine them vividly enough upon thinking about them to physically feel nauseous. Victoria finds viewing photographs of herself particularly disturbing as they reinforce her perceived view of her appearance due to them being thought of as concrete objects that truly represent the subject matter they show: “it makes me worry because I must look that vile in real life.” This then leads her to attempt to localise the features that cause her disgust, but instead, she identifies new areas of concern in the process, and this torturous cycle continues.

## Discussion

This section will attempt to theorise the self-identified origins of participants’ BDD by drawing on phenomenological concepts and psychological literature. We will demonstrate how a phenomenological perspective can be valuable in helping us gain a richer understanding of possible origins or contributing factors towards the development of BDD from individuals who live with the disorder and potentially aid their care and treatment. In the remainder of the discussion, we will draw on the work of Merleau-Ponty, a phenomenologist known for his contributions to understanding bodily perception and consciousness. We will also draw on Sartre and Heidegger’s work to reflect upon issues of interacting with the world and existentialism.

The data presented in this study illustrate the varied ways that individuals living with BDD believe the disorder developed for them, ranging from experiencing appearance-focused bullying, rejection, shame, sudden onset and the solidifying over time of perceived defects. To make sense of the events contributing to the development and onset of their BDD, participants employed imagery, for example, of being attacked by words, feeling strong bodily sensations and vivid descriptions of self-surgery, for example.

Due to the body being affected by the events described by participants, it becomes a focal aspect of their experience as the development and manifestations of the disorder are lived through their bodies. Very often, the body is treated as a biological object in psychology, but the body is also a social object (the means by which our identities are ‘read’ by others) and a perceiving object (the means by which we understand the world, and the people in it). Merleau-Ponty has written in great depth about embodiment in relation to perception and the social world. He suggests that our bodies are central to our ability to experience and understand the world and are interwoven with our perceived world (Merleau-Ponty, 1945/1962).

Through a phenomenological perspective, we are able to access and pay attention to the subtle nuances of individual phenomena expressed by participants and better understand from where and how the onset of BDD may originate. Living with a disorder such as BDD can greatly affect an individual’s ‘being-in-the-world’ (Heidegger, 1927/1962), as the relationship with one’s body is disrupted and the interface with one’s world is affected.

The disruption of a participant’s being-in-the-world is exemplified by Angelina’s account of experiencing appearance-focused bullying. Bullying was identified as a potential contributing factor of BDD development by several participants in our study. In addition to this, Weingarden et al. (2017) found that bullying was the most commonly prevalent experience reported by BDD patients, further suggesting that receiving criticisms of appearance may be linked to BDD onset. Having many parts of Angelina’s appearance extensively criticised and mocked by her peers may have directed her concerns on those aspects and therefore changed the way she views her existence in the world. This situation is reminiscent of being *othered* (Wilkinson and Kitzinger, 1996), as she is made to feel inferior to her peers, reinforcing her perception that she is different to the larger social group. Similarly, Elliot may also have experienced othering when his moles were pointed out by the children in his class. This interaction led him to make the decision to engage in self-surgery in an attempt to remove the moles from his body using “a sharp stone in the playground,” thus removing the very object that caused him to be othered in that situation. Elliot taking the stone and using it as a tool to alter his body could be described as a fulfilment of Heidegger’s (1927/1962) concept ‘readiness-to-hand’ which “captures the serviceability or usability connotations that belong to the very being of implements” (Stapleton, 2010, cited in Davis, 2010: 51). Elliot’s motivation to radical self-deforming action conveys his strong desire to change his body. He viewed the stone as a tool that could help him remedy the problem he had with this particular aspect of his appearance, and the use of such an unsuitable item highlights the sense of agonising desperation he felt in that exact moment. Elliot’s altering of his appearance could also be likened to him viewing his body as a ‘body project’. Placing emphasis on one’s body and adapting our appearance for public display turns our body into a ‘project’ (Nettleton and Watson, 1998: 1). Shilling (1993) states that “the body might best be conceptualised as an unfinished biological and social phenomenon, which is transformed, within limits, as a result of its participation in society” (Shilling, 1993, cited in Nettleton and Watson, 1998: 7). When viewing Elliot’s body as a project, we can consider the possibility of him altering the appearance of his body due to his want to be accepted as part of his social group and being objectified under their critical gaze.

Sartre noted that, as humans, we become aware of ourselves when we are viewed by others. It could be due to this sense of ‘bodily vulnerability’ (Dolezal, 2017: 423) that Elliot became more aware of his moles during the event he describes. The fact that he attempted to change a part of his physical appearance subjected to critical gaze suggests that, from this moment, he was no longer unconscious of his appearance (being-in-itself; Sartre, 1943), but now aware of his own appearance and others’ perception of his appearance, and wanting to adjust it in response to the criticisms (being-for-itself; Sartre, 1943). In psychological literature, there is some evidence to suggest that a number of people with BDD ‘dissociate’ or feel betrayed by their bodies. For example, they may experience thoughts such as “‘the way my face/body looks made this happen to me. My body betrayed me therefore I hate my body’” (Constantian, 2019: 119). It could be said that some people with BDD who receive criticisms about their appearance from others in the same way that Angelina and Elliot have may feel betrayed by their appearance and resort to self-surgery or self-harm. The behaviour that Elliot describes is in line with findings from Veale’s (2000) study, in which he highlights the fact that people with BDD may engage in self-surgery, or ‘DIY surgery’, in order to alter their appearance without going to a surgeon. This often has disastrous effects on the individual and can worsen BDD symptoms.

Rejection, shame and a sense of not being good enough were fundamental experiences to participants living with BDD. Previous literature has explored the relationship between shame and BDD (Weingarden et al., 2018); however, the personal lived experience of participants was not explored. Instead, participants’ shame was measured using the Young Schema Questionnaire-Short Form. In Weingarden et al.’s (2018) study, shame was presented as an internal emotion experienced by people living with BDD as a result of their BDD and concerns about the way others may view their perceived flaws. However, it did not address other potential sources of external shame such as bullying and rejection, which played important roles in the lives of participants in this study. As a consequence, we argue that future studies of BDD should incorporate clear and robust methods for thinking about the dimension of shame. Sartre described shame “as central to the ontology of human existence” (cited in Dolezal, 2017: 421). All participants reported several events that led them to feel a sense of shame. In most cases, the events were directly linked to the participants’ physical appearance, but there were some exceptions.

Heidegger (1927/1962) put forward the idea that we often take our bodies for granted, and only become aware of our bodies when we become ill or experience a feeling of pain or discomfort. It is possible that being subjected to an external source of shame may lead a person to become aware of bodily concerns. However, a person with BDD may perceive this critiqued image of their self as being concrete and completely real to them, thus reinforcing their belief that their appearance is defective. This may have been the case for Claire, who stated that she only saw individual “faults” rather than her whole face when she viewed herself in the mirror after experiencing rejection and criticism of her appearance. Victoria also talked about elements of her appearance transcending concern and becoming ‘deformities’ for her. It was noted by Veale and Riley (2001) that as individuals with BDD looked in the mirror, they became increasingly self-conscious and their negative perceptions of themselves were reinforced, which further elucidates Claire’s and Victoria’s experiences. Further to this, a clinical eye-tracking study by Greenberg et al. (2014) examined attention bias in people with BDD and a healthy control group and found that the BDD group focused on features on their own faces that they considered unattractive when viewing photographs of themselves. This finding is in line with participants from our study who often reflected on being preoccupied by specific areas of concern in their appearance when viewing themselves in mirrors or photographs. These areas of concern were often linked to criticisms made by others.

As well as participants receiving criticisms of their appearance, non-appearance related criticisms were also of interest. The experience of being criticised for behavioural aspects of the self was talked about by Jenny and Rohit, who both recalled receiving physical or verbal abuse from family members at a young age. Recent findings from Constantian (2019) suggest that childhood abuse may be linked to the experience of feeling ashamed of one’s body, as it ‘imparts a sense of vulnerability, fear, and helplessness’ (Constantian, 2019: 139). In addition, it has been argued that all types of abuse are tied to the body ‘by inflicting fear or pain, denying needs, or negating the importance of the individual’ (Herman, 1992, cited in Constantian, 2019: 140).

Participants additionally reflected on what their self-identified BDD triggers were at the time of interview and we felt this was an important step towards finding out what may contribute towards or worsen BDD symptoms. The triggers were varied, and all posed a substantial threat to the wellbeing of the individual. Angelina’s triggers include crossing paths with people from school and viewing photographs of herself that she does not like. This example is reminiscent of embodied knowing (Merleau-Ponty, 1945/1962), in which the world foregrounds the body. In this instance, elements of the external world (people from Angelina’s school or certain photographs) draw Angelina’s attention to the parts of her body she is affected by as a result of reminders of the harsh criticisms she faced and still associates with them, in a similar way that knowledge and experience can be ‘imprinted’ on one’s body (Tanaka, 2011, cited in Stenner et al., 2011: 149). She alludes to a cyclical relationship between experiencing BDD thoughts and triggers, making it difficult for her to separate the two and establish what is worsening her BDD. Weingarden et al. (2017) found that participants in their study ‘actually recalled an early experience of a BDD ritual, rather than the trigger of their BDD onset itself’ (Weingarden et al., 2017: 23), highlighting the difficulty in differentiating rituals and triggers. However, not all participants named specific triggers that worsened their symptoms. In addition, the rumination that Angelina describes may be further explained by a finding from Osman et al.’s (2004) study, which stated that people living with BDD who were bullied often experience spontaneous memories of the bullying and teasing that took place.

It is clear from the participant data that BDD is all-consuming and affects the lifeworld of those who live with it. As Bethany states, *“it* [BDD] *is there with me constantly every day.”* She acknowledges that it is a part of her life, that is, she lives with it and is forced to adapt her being-in-the-world in order to accommodate it.

## Conclusion

This study explored the self-identified origins of Body Dysmorphic Disorder (BDD) in eight participants living with BDD and how the disorder impacts their daily lives using Interpretative Phenomenological Analysis (IPA). The study identified and made sense of some potential contributing factors towards the development of BDD, such as experiencing childhood bullying and criticism, rejection and abuse. We also explored the idea of differing rates of BDD onset ranging from gradual development to sudden onset.

The misunderstandings and stigma attached to BDD are damaging to those who live with it and, in facilitating a more accessible way for individuals to display facets of the disorder, it is hoped that this study can help to raise the profile of BDD, help people become aware of its manifestations and reduce the surrounding stigma and ambiguity presently associated with it. Although there were similarities in the experiences participants shared across cases and across the study’s main themes, the way each participant made sense of those experiences was entirely unique and thus provided a variety of perspectives on multiple aspects of the disorder, made possible by IPA’s focus on the idiographic. There is still much more to learn about the origins of BDD, therefore more research in this area is needed in order to bring its potential beginnings, symptoms and manifestations to the attention of professionals and aid them in providing treatments and necessary support to those living with BDD. We believe using qualitative approaches such as IPA can help to gain a deeper understanding of what it is like to experience BDD from the perspective of those with lived experience of the disorder. Phenomenological research could also provide further insight into under-researched areas such as implications of rate of BDD onset, investigations into coping strategies and management of BDD symptoms, experiences of help seeking and the effectiveness of treatments for BDD. To conclude, we wish to highlight the need for professionals working with those affected by BDD to take into account the role of problematic social-developmental events, interpersonal relations and emotions with regard to how people understand their own BDD onset, moving away from previously held assumptions about the disorder. Additionally, we emphasise the importance of continuing to foreground the experiences of people living with BDD, as they currently remain vastly unheard and underrepresented in the research domain.

## Data availability statement

The datasets presented in this article are not readily available because of ethical reasons. Requests to access the datasets should be directed to S-LC, s.craythorne2@aston.ac.uk.

## Ethics statement

The studies involving human participants were reviewed and approved by Aston University Health and Life Sciences Ethics Committee. The patients/participants provided their written informed consent to participate in this study.

## Author contributions

S-LC: conceptualisation, methodology, investigation, and writing–original draft. RS and ML: supervision and writing–review and editing. All authors contributed to the article and approved the submitted version.

## Conflict of interest

The authors declare that the research was conducted in the absence of any commercial or financial relationships that could be construed as a potential conflict of interest.

## Publisher’s note

All claims expressed in this article are solely those of the authors and do not necessarily represent those of their affiliated organizations, or those of the publisher, the editors and the reviewers. Any product that may be evaluated in this article, or claim that may be made by its manufacturer, is not guaranteed or endorsed by the publisher.

## Footnotes

^1^ www.bddfoundation.org
